# Does Academic Medicine Matter?

**DOI:** 10.1371/journal.pmed.0030340

**Published:** 2006-09-12

**Authors:** Jocalyn Clark, Peter Tugwell

## Abstract

The authors discuss a new systematic review that found no difference in health outcomes between academic and nonacademic health care settings.

Academic medicine occupies a spot in that rarefied world in which one doesn't have to do much to justify one's existence. The best health care, the best research, the brightest minds on the planet circulate within academic medicine, together conspiring for the benefit of patients and practitioners.

Correct? Perhaps not, as growing concern about a decline in the state of academic medicine around the world threatens this taken-for-granted position [1–6]. In the past few years more and more critics have questioned the role and contribution of academic medicine—to society, to health-care systems, and to patients [7]. Who is academic medicine for? Why does it matter? What added value does it bring to disease prevention, health promotion, and the practice of health care? In order to engage our stakeholders—which include policymakers and the public—we need to demonstrate the benefits of academic medicine.

Revitalising academic medicine matters because health costs and expectations are rising alongside growing concerns that academic medicine is failing to produce sufficient gains for patients and funders. As others have argued, it is time for academic medicine to step up to the plate—to take leadership in redressing the gaps between bench and bedside medicine [8] and between the rich and the poor [9,10]. Taking leadership involves better preparation for future health-care demands in terms of attracting suitable and sufficient human resources, and aligning priorities with global health needs.

That said, however, the case for why and how academic medicine ought to lead us forward must be evidence-based. At a time when there is increasing demand for academic medicine to be more accountable, we find that the evidence base for its effectiveness is rather slim. Fortunately there is mounting interest in evaluating the impact of academic medicine across the traditional triad of medical research, education, and clinical care.

For example, in May of this year, the Academy of Medical Sciences released its report on the benefits of medical research to society (http://www.acmedsci.ac.uk/images/project/Medicalr.pdf). The Association of American Medical Colleges (http://www.aamc.org) has led work on improving the quality of medical education for the next generation of doctors and other health practitioners. And in a new systematic review in *PLoS Medicine*, John Ioannidis and colleagues examine the benefits of academic health care in terms of clinical outcomes [11].

## Evaluating the Evidence

The International Campaign to Revitalise Academic Medicine (ICRAM), of which we are cofounders (among others), was launched in part to evaluate the evidence base of academic medicine [12]. John Ioannidis is a member of the working party of ICRAM and with his colleagues undertook a systematic review of studies evaluating the outcomes of care in teaching hospitals. Previous work has produced mixed findings, and Ioannidis and colleagues' work may be the first attempt to systematically review the international literature. Their study is a valuable exercise; it not only provides a summary of the impact of academic versus nonacademic health care, but also identifies deficiencies in the current evidence base.

Ioannidis and colleagues posed the question, Compared to non-academic health-care structures (hospitals, clinics, systems), do academic teaching hospitals produce better or worse patient outcomes? Their findings suggest that, contrary to what many would expect (or hope), there is no evidence that teaching hospitals differ from nonacademic ones.

The researchers reviewed 132 studies and found virtually no differences in mortality outcomes between teaching and nonteaching health-care structures across a range of diseases, even after adjusting for confounders. That is, they found no benefits in terms of mortality for patients receiving academic health care, but they also found no harms from receiving such care.

The merits of this study lie firstly in its broad scope and systematic approach. Previous reviews have been unsystematic and none to our knowledge has shown the range and quality of searching of Ioannidis and colleagues' study. Secondly, the study includes careful statistical adjustment for confounders. A chief concern in this area has always been the ability to account for factors such as case mix. After all, teaching hospitals tend to treat sicker patients and trainees need to learn by doing. In the other direction, teaching hospitals have more advanced technology and see a high volume of cases that is often associated with better outcomes [13]. Ioannidis and colleagues included only adjusted estimates in their model, and specified whether these were for volume, severity, or comorbidity. In a sensitivity analysis that looked at only those comparisons in which all three confounders were accounted for, still no significant differences were found between teaching and nonteaching health care.

As the authors point out, the literature is patchy and diverse, made up mostly of nonrandomised studies, and focused largely on “hard” patient outcomes such as mortality rather than “softer” patient reported outcomes such as distressing symptoms (pain), disability, and satisfaction. Randomising patients to receive academic versus nonacademic care (whether by provider, clinic, or system) is obviously a logistical challenge. Observational data remain far less ideal because they are prone to bias.

Similarly, it is unfortunate that the systematic review fails to shed light on outcomes for specific subgroups of patients. As part of new social accountability initiatives, some teaching hospitals focus especially on disadvantaged groups such as the indigent populations, and we do not yet know whether academic health-care settings generate better outcomes for these or other groups.

Additional limitations emerge from the fact that the group of studies included in the systematic review is heterogeneous (though the authors account for this to the extent that they can), and undoubtedly residual confounding remains. In addition, almost three-quarters of studies included in the systematic review were conducted in the United States, and these are hardly generalizable to low- or middle-income countries. These US studies are even difficult to apply to other industrialized countries' health systems.

## Engaging Our Stakeholders

Ioannidis and colleagues' work reveals important defects in the primary literature that limit our attempts to sell the story of academic medicine to our stakeholders. Most studies so far provide unadjusted data and define a narrow set of outcomes, and very few have been conducted outside of the US. It appears few, if any, evaluations of academic medicine have been done in developing countries. While many of the challenges facing the future of academic medicine are shared by rich and poor countries [14], there are unique features of developing economies that merit special attention. Ioannidis and colleagues' systematic review takes stock of the existing evidence regarding which aspects of academic health care work and which do not, and for whom, and paints a small picture. Clearly, in an era of changing expectations and demands for accountability, the systematic review signals the need for better and broader research methods, both quantitative and qualitative.

Ioannidis and colleagues' study is an important step forward, and should be a call to arms for us all to work together to develop an evidence base on the outcomes of academic medicine.

## Abbreviations

ICRAM: International Campaign to Revitalise Academic Medicine
